# Towards Real-Time and Rotation-Invariant American Sign Language Alphabet Recognition Using a Range Camera

**DOI:** 10.3390/s121114416

**Published:** 2012-10-29

**Authors:** Hervé Lahamy, Derek D. Lichti

**Affiliations:** Department of Geomatics Engineering, The University of Calgary, 2500 University Drive N.W., Calgary, AB T2N 1N4, Canada; E-Mail: ddlichti@ucalgary.ca

**Keywords:** posture recognition, range camera, segmentation, tracking, 3D signature, rotation invariance, accuracy assessment

## Abstract

The automatic interpretation of human gestures can be used for a natural interaction with computers while getting rid of mechanical devices such as keyboards and mice. In order to achieve this objective, the recognition of hand postures has been studied for many years. However, most of the literature in this area has considered 2D images which cannot provide a full description of the hand gestures. In addition, a rotation-invariant identification remains an unsolved problem, even with the use of 2D images. The objective of the current study was to design a rotation-invariant recognition process while using a 3D signature for classifying hand postures. A heuristic and voxel-based signature has been designed and implemented. The tracking of the hand motion is achieved with the Kalman filter. A unique training image per posture is used in the supervised classification. The designed recognition process, the tracking procedure and the segmentation algorithm have been successfully evaluated. This study has demonstrated the efficiency of the proposed rotation invariant 3D hand posture signature which leads to 93.88% recognition rate after testing 14,732 samples of 12 postures taken from the alphabet of the American Sign Language.

## 1. Background and Objective

Interactions between humans and computers are typically carried out using keyboards, mice and joysticks. In addition to being different from the natural human way of communicating, these tools do not provide enough flexibility for a number of applications such as manipulating objects in a virtual environment. In order to improve the human-computer interaction, an automatic hand gesture recognition system could be used. Hand gesture recognition is the process by which gestures made by the user are automatically recognized in real-time by computer software via a camera. Hand gesture recognition has gained popularity in recent years, and could become the future tool for humans to interact effectively with computers or virtual environments.

Extensive research has been conducted in the field of gesture recognition in recent decades. Though very high recognition rates are usually claimed by authors who have used a variety of techniques (100% for [1], 98.6% for [2], 98% for [3]), hand gesture recognition remains a timely research topic with many unresolved problems. This can be seen when taking into account the high number of papers written on the topic in 2012: more than 60 were found using only the Compendex database with a query made on 16 April 2012. Gesture recognition is performed most frequently through supervised classification processes where different features are used to predict the class membership of the considered hand image. The reference gestures are stored in a database and during a subsequent real-time image acquisition, the current gesture is matched with the most similar one available in the training dataset. To perform the classification, a huge number of classifiers such as neural networks, support vector machines, graph matching, inductive learning systems, voting theory, hidden Markov models, chamfer distance or dynamic Bayesian networks are used. Extensive training and testing are performed after acquisition of a high number of datasets from multiple users. A confusion matrix is generally presented to show the success rate. In most of the cases, a recognition rate over 98% percent is presented, but with a limited number of gestures acquired under specific conditions.

Most of the published literature on hand gesture recognition doesn't consider using the advantages that a 3D signature can provide. For example, in [4], after generating a point cloud of a hand posture from data captured with four web cameras, the authors use cylindrical virtual boundaries to randomly extract five slices of the point cloud. Each slice is processed by analyzing the point cloud distribution and the hand posture is recognized from this analysis. By doing so, though the hand postures are represented by a 3D point cloud, the full 3D topology is not considered in the recognition process. Other researchers, though using a 3D sensor, do not consider at all the third dimension in the features used to represent the hand postures. That is the case of [5], where the authors use a 3D depth camera but only consider the 2D outline of the hand segment in their recognition process. A 3D image describes better the hand posture than a 2D image. It provides less occlusion. The 2D image is the projection in a given plane of the 3D image. Two different 3D hand postures projected on a particular plane can provide exactly the same information in the 2D images which as a consequence cannot be used to differentiate them.

The design of a rotation invariant system has not been successfully achieved so far. Indeed many researchers consider the principal component analysis to evaluate the orientation of the 2D hand image but, as acknowledged by [6], this method is not always accurate. Not only has the estimation of the rotation of a 2D hand segment not been successful so far but, furthermore, the evaluation of the orientation of a 3D hand segment is not considered in most existing approaches.

To test their hand motion classification using a multi-channel surface electromyography sensor, [7] only consider five testing images per gesture. Contrary to most of the studies on this topic, a significant number of testing samples has been considered to validate the proposed algorithm. Indeed, testing more than 1,000 images per gesture in average instead of five provides more evidence on the robustness of the methodology.

The objective of the current study is to design a range camera based system where a high number of postures taken from the alphabet of the American Sign Language can be recognized in real-time. Contrary to existing methods, the current one allows hand posture recognition independently of the orientation of the user's hand. It makes use of a 3D signature, considers only one training image per posture and uses a significant number of testing images for its evaluation.

The term “gesture” means that the character considered cannot be performed without a dynamic movement of the hand while “posture” refers to a character that can be fully described with a static position of the hand. In this paper “Static gestures” doesn't mean that the user is not moving his hand. “Static gestures” or “postures” relate to the different characters of the American Sign Language Alphabet as shown in the paper except “Z” and “J”. Once the user performs one of them, he can rotate and move his hand in whatever direction he wants to. The objective is to make the system recognize this posture no matter the position of the user's hand.

This paper is structured as follows: Section 2 reviews the literature on methods used for hand gesture recognition. Section 3 describes the set up of the experiment and Section 4, the segmentation process used. The methodologies considered for tracking the hand motion are provided in Section 5. Section 6 reports on the evaluation of the segmentation as well as the tracking processes. In Section 7, the recognition principle is depicted. The rotation invariance algorithm is highlighted in Section 8. The experimental results and their analysis are shown in Section 9 while a comparison with results from other papers is discussed in Section 10. The conclusion and future work are provided in Section 11.

## 2. Literature Review

Before describing the different steps of the algorithm applied herein, a literature review has been conducted on the methods frequently used regarding hand posture recognition. The review has focused on the input data, the sensors, the segmentation as well as the tracking processes, the features used to represent hand postures and finally the classifiers. Most of the papers selected are dealing with the American Sign Language recognition. This section ends with the limitations of current method hence the need of going further in this research and highlights the remaining structure of the current paper.

### 2.1. Input Data

Most of the research conducted in the field of gesture recognition makes use of 2D intensity images acquired as snapshots or at video rates [8]. In very rare cases, 3D data are obtained from stereo images [9]. Range data extracted from color images after analysis of the deformation of the patterns on object surfaces is used by [10] while [3] consider depth information obtained from a range camera.

### 2.2. Sensors

Different sensors have been used to improve the interaction between man and machine. While [11] uses the infrared time-of-flight range camera, the Logitech Messenger Webcam is the sensor considered by [12]. A camera that provides a stereo pair of images is used by [13]. Most researchers suggest natural interaction without any additional equipment to the user's hand, but others make use of specific gloves ([1,10]) or markers to derive meaningful results. In [14], the images of the user's face and hand were acquired with a service robot. Most recently, some 3D sensors such as the Microsoft Kinect [15] and the Leap sensor [16] are currently being used by some researchers for the same purpose of improving interaction with computers by using hand gestures.

### 2.3. Extraction of Region of Interest

In order to recognize the hand gesture, the hand information must first be extracted from the acquired images. Different approaches are available in literature to achieve this process, called segmentation.

The most commonly used technique for hand segmentation is color-based, as demonstrated in [17]. The skin color is a distinctive cue of hands and is invariant to scale and rotation. Human hands have almost the same hue and saturation but vary in their brightness. This method was performed by [12] by analyzing the red and green components of the skin color in the red, green, and blue color space which is then converted to the hue, saturation and intensity color space (complete range of colors that can be displayed and recorded on digital video) as the intensity component can be treated separately from the chrominance components.

Another method is based on image differencing between consecutive video frames [18]. A hand gesture detection and segmentation method is proposed where video sequences are captured from a stationary camera with complex backgrounds. The hand segment is extracted based on a threshold grey value calculated from the image's intensity histogram. In [19], the hand motion is detected using double difference of range images.

To extract the hand information from a range image obtained from an active time-of-flight camera [20] make use of depth constraint to separate the foreground and the background of the image. The major cluster in the image at a distance smaller than a pre-defined threshold value can be treated as the hand. A simple depth keying is also used by [11] to define the region of interest. In [10], an initial segmentation is obtained by the means of thresholding the depth values. The segmentation of the arm is achieved by a hierarchical unsupervised clustering procedure. It is an iterative process where clusters are defined and merged based on Euclidean distance. To segment the hand from the forearm, the method used is based on a statistical modeling, the idea being to classify the point cloud based on a probability distribution modeled as a mixture of Gaussians. Experimental results demonstrate robustness of the algorithm under various orientations of the palm and fingers. In [2], 3D information is used for segmentation and detection of face and hands using normal Gaussian distribution and depth information.

A robust segmentation technique based on fusion of range and intensity images using a time-of-flight camera is proposed by [21]. According to the authors, none of the intensity, range and amplitude data delivered by the camera for every pixel can be used alone to make robust segmentation under variant lighting conditions. The proposed method is based on the combination of two unsupervised clustering approaches: K-means and expectation maximization. K-means clustering is a method of cluster analysis which aims to partition n observations into k clusters in which each observation belongs to the cluster with the nearest mean. An expectation-maximization algorithm is a method for finding maximum likelihood estimates of parameters in statistical models, where the model depends on unobserved latent variables. They both attempt to find the centroids of natural clusters in the fused data. The K-means algorithm is used to define the initial clusters' centers, which reduce the sensitivity of the initial points. These initial parameters are then used by the expectation maximization technique to find the sensitivity of the local maxima. The experimental results show that the proposed gesture segmentation technique can successfully segment the hand from user's body under variant illumination conditions in real time. This idea of fusing range and intensity images for image segmentation was also considered by [22]. The assumption is that pixels with similar 3D coordinates and similar intensities belong to the same physical object. The 3D position and intensity are then combined into a 4D intensity/position feature space. The object-based segmentation is then performed by clustering pixels in the new intensity/position feature space. A divisive clustering method without any prior knowledge has been considered where the cluster with the highest variance is split into two using a hyperplane.

### 2.4. Hand Segment Tracking

The segmentation processes described in the previous section applied on every acquired image is time consuming. In order to avoid this situation and to enable a real-time application, the hand cluster is segmented from the first image acquired and tracked through the subsequently acquired images.

The Kalman filter is the most commonly used technique for tracking moving objects over time. While tracking independent clusters [22] assumes the motions along the X, Y and Z directions to be decoupled and therefore predicted by separate Kalman filters. The motion of the clusters is assumed to have constant velocity. To account for slight changes in the velocity, the continuous-time acceleration is modelled as white noise. The parameters of the filter are the process noise and the measurement noise [23] make use of the Kalman filter to predict the hand location in one image frame based on its location detected in the previous frame. The Kalman filter in [23] is used to track the hand region centroid in order to accelerate hand segmentation and choose the correct skin region. Using a model of constant velocity motion, the filter provides and estimates the hand location, which guides the image search for the hand. Unfortunately, in most of the papers published on this topic, no detail is provided on how the process noise and the measurement noise are estimated. Similarly, the Kalman filter is not evaluated and its limits for hand motion tracking are not known.

Another technique used for tracking a hand segment within acquired images is the condensation algorithm. In [24] its authors argue that trackers based on Kalman filters are of limited use because they are based on Gaussian densities which are unimodal. They suggest the condensation algorithm which is highly robust in tracking agile motion in the presence of dense background clutter. The condensation algorithm (conditional density propagation) allows quite general representations of probability. One of the most interesting facets of the algorithm is that it does not process every pixel of the image. Rather, pixels to process are chosen at random, and only a subset of these pixels ends up being processed.

The mean-shift method is a powerful and versatile, non-parametric and iterative algorithm that has been used for tracking hand motion. For each data point, the mean-shift algorithm associates it with the nearby peak of the dataset's probability density function. The mean-shift defines a window around it and computes the mean of the data point. Then it shifts the center of the window to the mean and repeats the algorithm till it converges. After each iteration, the window shifts to a denser region of the dataset. At the high level, the mean-shift algorithm can be summarized as follows: fix a window around each data point, compute the mean of data within the window and shift the window to the mean and repeat till convergence. The classic mean-shift algorithm is time intensive. Many improvements have been made to the mean shift algorithm to make it converge faster. This method has been used by [13] in association with the Kalman filter.

### 2.5. Features Used to Represent Hand Postures

The recognition of the hand gesture is performed not by using the raw image information but rather by representing it by some different types of parameters known as signatures or features. Geometric features such as fingertips [25], finger directions, image moments [26], orientation histograms [27], depth information, hands' contours [28], and labeled graphs [29] are commonly used to recognize the pattern of a hand gesture. Principal component analysis has been used by [10] to extract feature vectors by defining an orthogonal space and projecting an image into that space. Hand shapes and distance transformation images are used by [3] to represent hand gestures. A combination of statistical features made of the seven Hu-moments derived from second and third order moments and geometric features such as rectangularity and circularity has been used by [2]. Recognizing gestures not only needs spatial features, but also requires temporal features such as the hands' positions, velocities and trajectories [30]. Most of these features vary depending on the lighting conditions, the size of the hand in the image, the orientation of the hand and the hand's position. For a robust gesture recognition algorithm, features independent of these elements are required.

### 2.6. Classifiers Most Frequently Used for Recognition

Classification methods or classifiers are used to identify every gesture. They make use of different techniques to match the current gesture with the most similar one available in the reference database. While hidden Markov models are considered by [28,29] employ the elastic graph matching technique to classify hand postures against complex backgrounds. A multivariate fuzzy decision tree was used by [31]. In [32], a semi-supervised fuzzy neural network is used for recognition of static gestures while hidden Markov models are applied for online recognition of dynamic gestures. In [33], the authors proposed to recognize hand gestures in a continuous video stream using a dynamic Bayesian network. The chamfer matching method is considered by [3] to measure the similarities between the candidate hand image and the hand templates in the database. In [19], a binary comparison of histogram bins results in a distance for each gesture used to perform the classification.

### 2.7. Conclusion

According to [34], further research in the areas of feature extraction and gesture representation is required to realize the ultimate goal of humans interfacing with machines on their own natural terms. Indeed, although [3] and others claim to have used 3D data, they mainly classify 2D images with the pixel values being the range information. An exhaustive description of hand gestures can only be made with 3D data, which is not currently widely used by researchers. In addition, the feature vectors used as signatures to represent the gestures appear to not be accurate enough to describe the hand gestures. Indeed, the equivalence between the original image and its representation which is the feature vector has not been proven in any paper. This absence of equivalence is the reason why most of researchers have been trying different feature vectors and different classifiers in order to come up with the best possible combination. As a consequence, further research has to concentrate on finding the best possible signature for every gesture. The same gesture performed by the same user or different users appear to show different orientations and scales. To overcome this problem, most of the researchers chose to increase the number of reference gestures stored in the database. As a consequence, the size of the database as well as the matching time increase and the number of gestures recognizable reduces especially when dealing with real-time applications.

**Figure 1.**
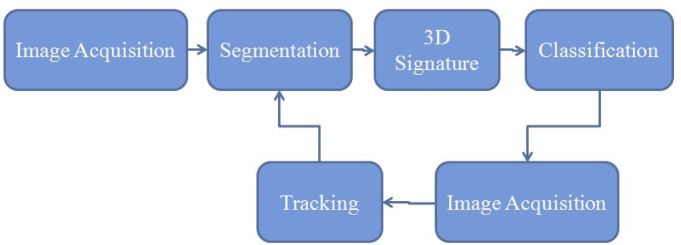
The proposed methodology for hand posture recognition.

The proposed method in the current paper (Figure 1) considers the 3D point cloud provided by a range camera to build a 3D model of the hand gesture and it makes use of only one single training image with the objective of showing the robustness of the method and also its appropriateness for a real-time application. To track the hand motion during the real-time process the Kalman filter has been proposed with a detailed explanation on how the process noise and the measurement noise have been modeled. The mean-shift algorithm has been tested as well. In order to achieve these objectives, the sensor considered is the SR4000 range camera because of its ability to provide 3D images at video rates.

## 3. Experiment Setup

**Figure 2.**
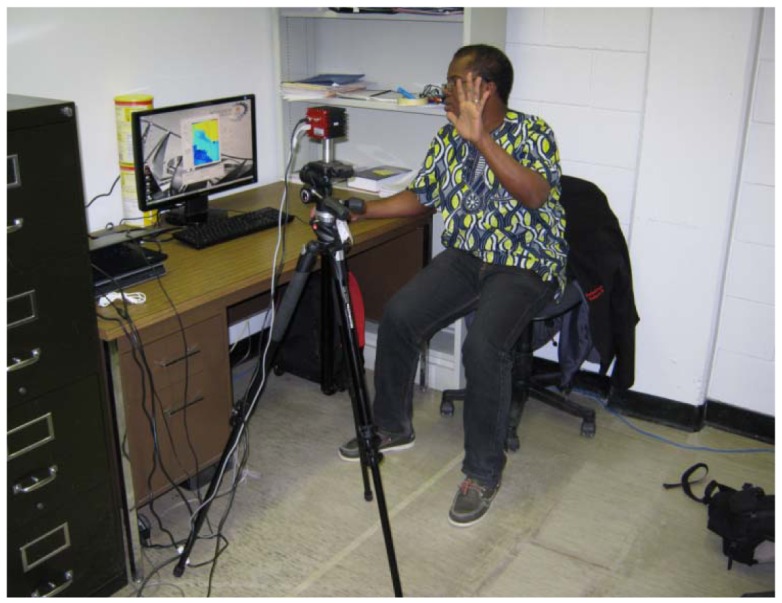
Experimental setup.

The hand posture recognition experiments have been conducted under laboratory conditions. The camera was mounted on a tripod situated approximately at 1.5 m from the user sitting on a chair and facing a desktop where the images acquired by the SR4000 camera as well as the results from the hand posture recognition are displayed in real-time. No particular background is required. Similarly, the user does not have to wear long sleeves or specific gloves as required in some similar experiments [10]. A picture describing the setup environment is shown in Figure 2. The integration time which is the length of time during which the pixels are allowed to collect light was set to 1.8 ms, corresponding to a theoretical frame rate of 43 images per second.

## 4. Segmentation

**Figure 3.**
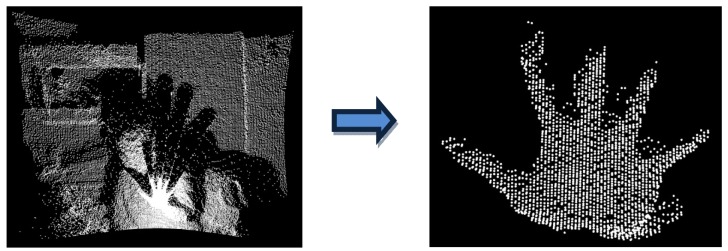
Objective of segmentation.

**Figure 4.**
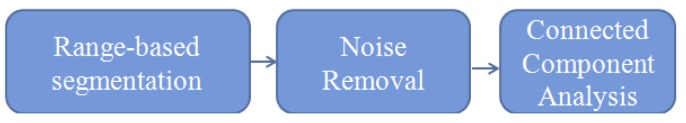
Multiple-step range based segmentation.

Segmentation is the process of grouping points that belong to the same object into segments. The idea here is to extract from the point cloud, the set of points that describe the user's hand (Figure 3). In this paper, a multiple-step range based segmentation has been designed (Figure 4).

### 4.1. Range-Based Segmentation

The underlying principle is that there shouldn't be any object between the camera and the hand. Thus the hand appears in the foreground of the image. The algorithm is designed based on the following two key points:

- Find the first 3,000 points closest to the camera using the range. This threshold was obtained from the analysis of the total number of points describing a hand posture with respect to the distance from the camera to the hand;

- Assuming that an average human's hand can fit within a 3D cube bounding box having 20 cm sides; a sub-selection is extracted to achieve this objective; the idea being to get rid of an eventual part of the user's arm.

**Figure 5.**
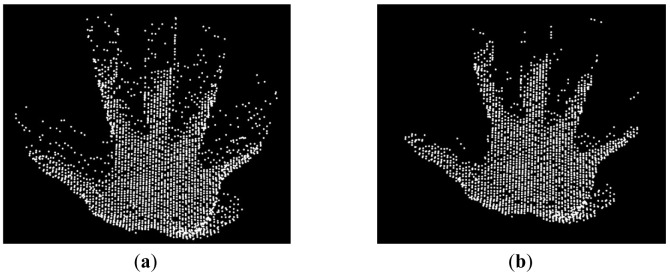
Result of the range-based segmentation Before noise removal (**a**); After noise removal (**b**).

An example of the result of this algorithm is presented in Figure 5(a).

### 4.2. Noise Removal

The results obtained contain the appropriate information but appear noisy due to the presence of hanging points (Figure 5(a)). The hanging points appear isolated compared to the ones that belong to the hand. The point density of the hand is much higher than the one of the hanging points. The point cloud obtained from the range-based segmentation is split into voxels (3D cells). Voxels that have a low point density are discarded from the segment.

The noise made of the hanging points is described in [35] as the result of lateral and axial motion artifacts of the sensor or the target object. A range image is obtained after averaging four different images acquired by the camera with 90° phase-shift and at different times. For each image, four samples of the reflected light are acquired and read out by the sensor. Lateral motion and axial motion of the hand with respect to the viewing axis of the camera during the acquisition of the four samples are the main cause of the noise (hanging points) in the image. Lateral motion results in the mixture of foreground and background phase values at the boundary of moving objects while axial motion describes motion along the viewing direction and introduce additional phase changes due to non-constant object distance. To remove this noise, the authors of [35] use the two raw images namely the shifted reference signal and the inverted signal, provided by the Photonic Mixing Device to compute the phase images. The objects are tracked in the latter in order to average corresponding pixels and a new depth image is computed after demodulation. The raw images used in this paper to remove the motion artifacts are not provided by the SR4000 and as a result this method which has the advantage of eliminating the noise by removing its causes cannot be applied in this research.

### 4.3. Connected Component Analysis

**Figure 6.**
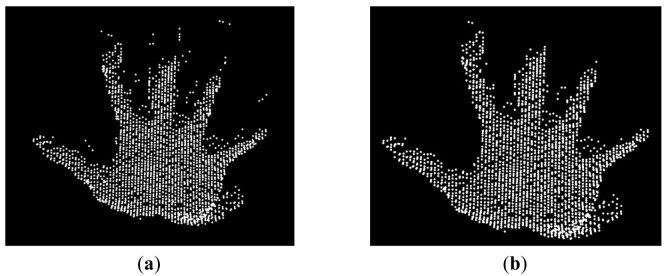
Remaining noise removal. Before using the connected component analysis (**a**); after using the connected component analysis (**b**).

This step is an additional noise removal algorithm. Connected component labeling is used to detect unconnected regions. It is an iterative process that groups neighboring elements into classes based on a distance threshold. A point belongs to a specific class if and only if it is closer within the distance threshold to another point belonging to that same class. After the noise removal, the hand segment appears to be the biggest one in the dataset. An example of the results from and connected component analysis is provided in (Figure 6).

The segmentation process made of the range segmentation, noise removal, and the connected component analysis operations is only applied on the very first image acquired. For the subsequent images acquired, only the noise removal steps are applied. In a real-time application, the segmentation is made only of noise removal from the region of interest obtained from the tracking process.

Every image acquired contains 25,344 3D points while every region of interest contains less than 3,000 points and in 90% of the cases, around 1,500 points. The segmentation process applied on every single image is time consuming. Indeed, the segmentation time of a dataset of 12,000 images has been reported on Table 1. The time of the different steps of the segmentation has been recorded in second. The computation has been performed using Microsoft Visual Studio and the time has been measured with the function clock. Table 1 shows that on average 3 s are required for an independent segmentation. As a consequence, a real-time processing cannot be achieved by segmenting systematically each time the whole image. Tracking the hand movement is therefore necessary.

## 5. Tracking

In this research, two tracking methods, namely the mean-shift algorithm and the Kalman filter have been tested and evaluated.

### 5.1. Mean-Shift Method

A simple version of the mean-shift algorithm has been implemented. The segmentation process described earlier is used to determine the initial coordinates of the centroid of the hand segment. A 3D cube bounding box of 20 cm side centered on this initialization point is then used to collect from the first frame the points expected to belong to the user's hand. Once selected, the centroid of the new set of points is determined. In order to identify the segment in the following frame, the newly determined centroid is considered as the center on the following hand segment and thus the center of the bounding box. Iteratively, hand segments are extracted and centroids computed. This method takes its advantage from the fact that the range camera provides the x, y, z coordinates of every pixel and for every frame in the same camera frame. In addition, because of the high frequency of the frames, it is assumed that the centers of the hand segments in two consecutive frames are quite close to each other in such a way that the centroid of the hand segment in the first frame can be used as centroid of the segment in the second frame. Consequently, this method applied iteratively enables a real-time tracking that is evaluated in Section 6.

### 5.2. Kalman Filter

Considering that the hand is undergoing a linear movement with constant velocity and applying Newton's law of motion, the linear discrete Kalman filter has been used for tracking the centroid of the hand. The state vector (*x*) comprises three states describing the position of the centroid of the hand segment in the camera frame (*p*) and three other states corresponding to the velocity of the hand movement (*v*). *p_x_*, *p_y_* and *p_z_* as well as *v_x_*, *v_y_* and *v_z_* correspond respectively to the position and velocity in *X*, *Y* and *Z* directions. The initial position coordinates are obtained from an initialization process where the user indicates approximately the starting position of the hand. An image of the hand is acquired and a segmentation process described earlier is applied to extract the hand segment from which the centroid is computed. The initial velocity is assumed to be null:

(1) $$\begin{array}{ccc} x=\left(\begin{array}{c} p\\ v \end{array}\right) & p^{T}=\left(\begin{array}{ccc} p_{x} & p_{y} & p_{z} \end{array}\right) & v^{T}=\left(\begin{array}{ccc} v_{x} & v_{y} & v_{z} \end{array}\right) \end{array}$$

The continuous-time-space state representation of the system is a first order vector differential equation given by:

(2) $$\dot{x}=Fx+Gu$$

*Fx* is the dynamics model and *Gu* the stochastic model. The dynamics model defines how the states evolve with time based on known physical relationships. The stochastic model is used primarily as a means of defining the uncertainty in the dynamics models where *G* is the shaping matrix. *w* = *Gu* is a vector of zero-mean, unity variance white noise. From its definition, the purpose of the stochastic model is not to model or characterize the specific variations in the states, but rather to capture their statistical properties after all of the systematic effects have been removed or accounted for. The assumption is that the underlying state vector is a random process. Based on the constant velocity model chosen:

(3) $$\begin{array}{ccc} F=\left(\begin{array}{ll} O_{3} & I_{3}\\ O_{3} & O_{3} \end{array}\right) & G=\left(\begin{array}{c} O_{3}\\ I_{3} \end{array}\right) & u=\left(\begin{array}{c} q_{{\dot{v}}_{1}}\\ q_{{\dot{v}}_{2}}\\ q_{{\dot{v}}_{3}} \end{array}\right) \end{array}$$

where *q_v̇_* is the power spectral density of velocity change and thus the white noise of the acceleration. *q_v̇_* does not depend on the sampling frequency. After collecting a sample dataset with the hand moving at a nearly constant speed, *q_v̇_* will be computed as the standard deviation (*σ*_a_) of the acceleration multiplied by the square root of the sampling frequency:

(4) $$q_{\dot{v}}=\sigma_{a}\sqrt{\frac{1}{\Delta t}}$$

The accelerations in *X*, *Y* and *Z* are computed as the corresponding differences of velocity divided by the elapsed time between three consecutive images. Similarly, the velocities are computed as differences of corresponding positions divided by the appropriate time. Accelerations can only be computed if a minimum of three consecutive positions of the hand centroid have been recorded. Once the time series of the accelerations is obtained, (*σ_a_*) is estimated as the standard variation of this set of accelerations.

The continuous-time system model presented above, although often convenient, is not well suited for the estimation of problems involving discrete time data. Therefore the continuous-time equation has to be converted into a corresponding discrete-time difference model of the following form:

(5) $$x_{k}=\emptyset_{k-1,k}x_{k-1}+w_{k-1}$$

where Ø is transition matrix that converts the state from epoch *k* − 1 to *k*. It is the discrete time equivalent of the dynamics matrix. *w_k_* is the process noise considered as white.

The transition matrix can be solved as follows:

(6) $$\emptyset_{k-1,k}=e^{F\Delta t}=I+F\Delta t+\frac{1}{2}F^{2}\Delta t^{2}+\ldots with\;F^{2}=O_{6}$$

(7) $$\emptyset_{k-1,k}=\left(\begin{array}{cc} I_{3} & \Delta tI_{3}\\ O_{3} & I_{3} \end{array}\right)$$

with Δ*t* being the time elapsed between the acquisition of the two consecutive frames *k* − 1 and *k*. The covariance *Q_w_* of the process noise can be computed as follows:

(8) $$Q_{k}=\int_{t_{k-1}}^{t_{k}}\emptyset_{k-1,k}GQ\left(t\right)G^{T}\emptyset_{k-1,k}^{T}\partial \tau$$

(9) $$Q_{k}=\left(\begin{array}{cc} \frac{1}{3}\Delta t^{3}q_{\dot{v}}^{2}I_{3} & \frac{1}{2}\Delta t^{2}q_{\dot{v}}^{2}I_{3}\\ \frac{1}{2}\Delta t^{2}q_{\dot{v}}^{2}I_{3} & \Delta tq_{\dot{v}}^{2}I_{3} \end{array}\right)$$

To update the state vector, the measurement (*z*) is made of the three position coordinates of the centroid of the hand segment:

(10) $$z_{k}=H_{k}x_{k}+v_{k}$$

(11) $$H=\left(\begin{array}{ll} I_{3} & O_{3} \end{array}\right)$$

where *H* is the observation matrix and *v* is the observation white noise that is assumed to be uncorrelated with *w*. The measurements errors are characterized by the covariance matrice *R. R* is obtained by considering that the observed coordinates have a precision of *σ* = 1 *cm* (Equation (12)). This value has been obtained by imaging a static object (Spectralon target) hundred times within similar set up conditions as for the hand posture recognition. For several pixels, the standard deviations have been computed in *X*, *Y* and *Z* directions of the camera frame. The variations for the central pixel range from 4 mm in *Y* direction to 10 mm in *Z* direction

The Kalman filter state prediction and state covariance prediction are computed as follows:

(12) $$R=\sigma^{2}I_{3}$$

(13) $${\bar{x}}_{k}=\emptyset_{k-1}{\hat{x}}_{k-1}$$

(14) $${\bar{P}}_{k=}\emptyset_{k-1}{\hat{P}}_{k-1}\emptyset_{k-1}^{T}+Q_{k-1}$$

where *x̂_k_* denotes the estimated state vector; *x̄_k_* is the predicted state vector for the next epoch; *P̂_k_* is the estimated state covariance matrix; *P̄_k_* is the predicted state covariance matrix.

The Kalman filter update steps are as follows:

(15) $$K_{k}={\bar{P}}_{k}H_{k}^{T}{\left(H_{k}P_{k}H_{k}^{T}+R_{k}\right)}^{-1}$$

(16) $$v_{k}=z_{k}-H_{k}{\bar{x}}_{k}$$

(17) $${\hat{x}}_{k}={\bar{x}}_{k}+K_{k}v_{k}$$

(18) $${\hat{P}}_{k}=\left(I-K_{k}H_{k}\right){\bar{P}}_{k}$$

where *K_k_* is the Kalman gain, which defines the updating weight between the new measurements and the prediction from the system dynamic model.

## 6. Evaluation of the Segmentation and Tracking Processes

**Figure 7.**
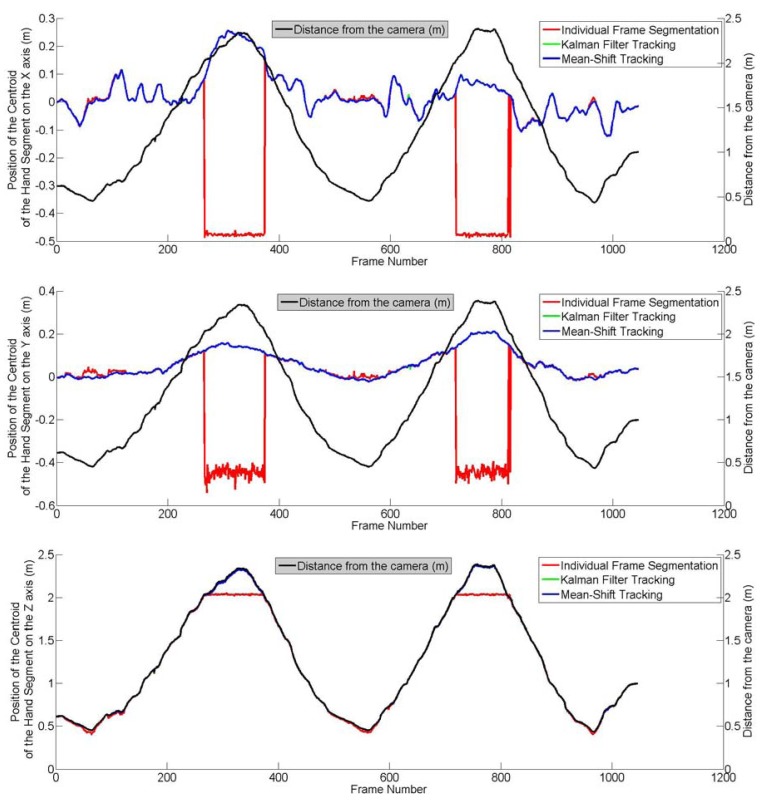
Evaluation of the segmentation and tracking process while varying the distance between the hand and the camera.

In this section, the assessment of the segmentation process as well as the two tracking processes are reported. In this assessment, the hand was moving back and forth (translation only) in the *X*, *Y* and *Z* directions of the camera. The movement of the hand is assumed to be linear with a nearly constant velocity in all three directions. In the case of the Kalman filter, any deviation from the assumptions of linearity and constant velocity are compensated by the process noise. During this experiment, 1,045 frames acquired have been saved. An individual segmentation was applied to each of them and the results used as ground truth. The mean-shift and Kalman filter algorithms were applied to the movement by considering the initial position of the hand to be the position of the centroid of the hand segment obtained from the first frame. Two parameters were considered in this evaluation: the distance between the camera and the hand and the speed of the hand movement, the objective being to check whether the accuracy of the tracking is a function of these parameters. As shown by Figure 7, the hand was moved away up to 2.3 m from the camera.

**Figure 8.**
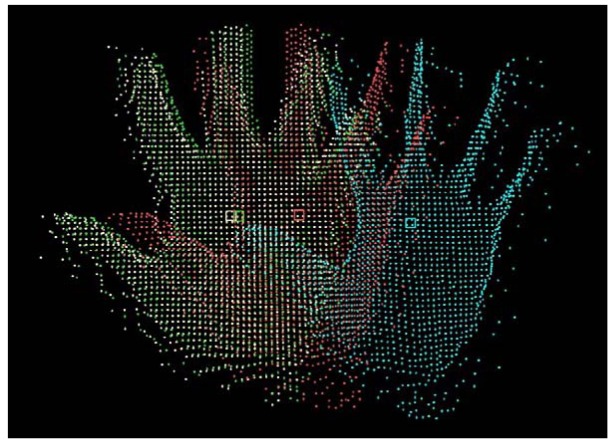
Tracking result: Tracked centroids and corresponding hand segments.

An example of the tracking process is shown in Figure 8 where the tracked centroids as well as the corresponding hand segments are displayed. Figure 7 shows three graphs where the position of the centroid of the hand segments have been plotted in the *X*, *Y* and *Z* axes of the camera frame. These graphs clearly show that the segmentation process, the Kalman filter algorithm and the mean-shift tracking algorithm give accurate results and are appropriate for hand motion tracking. However, it can be noticed on the graphs that when the distance between the hand and the camera exceeds 2 m, the results obtained from the segmentation process were wrong and that the coordinates obtained indicate a centroid much closer to the camera than the hand. The main assumption of the segmentation process is that there shouldn't be any objet between the hand and the camera (Section 4.1). The experiment was performed in a small and crowded room and this assumption couldn't be satisfied over 2 m which justifies the obtained results. The object automatically segmented in the frames 266 to 374 and in the frames 718 to 816 is not the user's hand.

The two tracking algorithms give similar results and match with the results from the segmentation process, which shows their appropriateness for hand motion tracking. The accuracy of the tracking methods have been computed by considering as true values the results from the segmentation process. The frames from which the object segmented is the hand have not been included in the computation of the root mean squares. Table 2 shows that this accuracy is sub-centimeter and that both methods are equally accurate. Another conclusion to be derived is that the quality of the tracking using either of these methods is independent of the distance between the hand and the camera.

The elapsed time between two consecutive acquired images including the image acquisition and the processing time is 28 ms on average, which corresponds to 35 images per second. This frame rate is good enough for a real-time visualization as 24FPS is the progressive format now widely adopted by film and video makers [36].

However, when moving the hand back and forth across the viewing direction of the camera at a velocity higher than 1 m/s (1.19 m/s for the Kalman filter and 1.33 m/s for the mean-shift) and on an approximate horizontal distance of 50 cm, it has been noticed that the designed methods fail to track properly the hand movement. The predicted position of the hand is no longer accurate. The considered methods are thus limited and in case of very fast movement of the hand (which is not the regular way for humans when moving their hands), further investigation is required.

## 7. Principle of Posture Recognition

**Figure 9.**
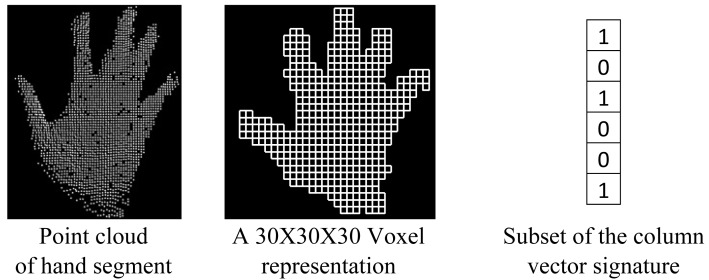
Generation of a hand segment signature.

Several commonly used recognition methods appearing in the literature have been described and evaluated in [37]. The maximum overall recognition rate obtained is 97.29% when using the outline of the 2D hand segment and its corresponding distance transformation image as features and the chamfer distance as classifier. But, unfortunately, these features are not appropriate for real-time application. In this research, the real-time recognition of the postures has been achieved by using an heuristic and voxel-based algorithm. To derive a signature of a hand posture from its point cloud, a 3D bounding box is generated and transformed into a regular grid. Only voxels containing at least one point from the hand segment's point cloud are considered in the hand posture's signature. A column vector is thus generated in which every voxel is represented by a Boolean value, true when it contains at least one point and false when it is empty. This vector contains the full 3D topology of the hand posture as voxels are stored following a predefined order. This signature is equivalent to the original posture as it can be used to reconstitute the 3D structure of the original posture. It considers the position and orientation of every single part of the hand segment. In addition, it has the advantage of containing less information and consequently it is easier to store and is more time-efficient for processing. It is a suitable parameter to measure the similarity between hand postures. With this signature, hand poestures are compared by considering their 3D structure. Figure 9 shows an example of the generation of a hand signature. A 30 × 30 × 30 representation has been considered because after testing empirically several combinations, it appears to be the one that provides the better results.

**Figure 10.**
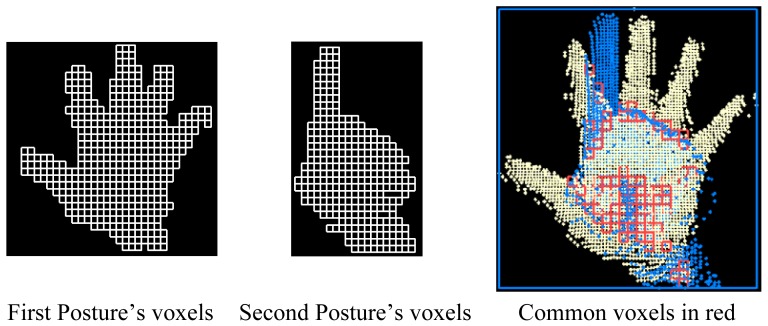
Methodology of comparison of two hand segments.

To compare two hand postures (Figure 10), one of the two point clouds is translated onto the second one by matching their centroids. The idea here is to define the same bounding box for both postures to be compared. The comparison is achieved by evaluating the percentage of similarity between the two postures in other words, the percentage of voxels containing at least one point in both datasets. This percentage is a suitable parameter to measure the similarity between hand postures. Thus, hand postures are compared by considering their 3D structure.

For each of the training images, the hand segment is computed and stored in addition to the corresponding class. For every image in the testing database, the posture recognition is performed by comparing the current posture to the training ones previously stored. The similarity measure between the candidate and all the templates in the training database is calculated and the highest score indicates the recognized posture. The selected posture is the one from the training database that is closest in 3D topology to the current posture. The classifier considered is thus the nearest neighbor.

## 8. Rotation Invariance

**Figure 11.**
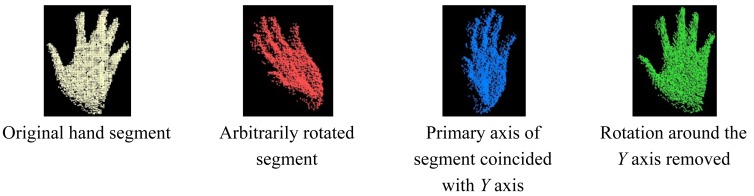
Example of rotation removal of a hand segment.

The performance analysis of different methods for hand posture recognition using range cameras [37] shows that none of the features considered are rotation invariant, which is not realistic for real-time applications as the users will not be allowed to rotate their hand while interacting with a computer. In this research, the rotation-invariance of the posture is achieved by measuring the orientation of the segmented point cloud and by removing that rotation before entering the recognition process. Once the orientation is removed, its signature can be computed. The evaluation of the orientation is achieved in two steps. Using the principal component analysis, the primary axis of the hand segment is derived by considering the eigenvector corresponding to the largest eigenvalue. The primary axis of a hand segment can be defined as the longitudinal axis that passes through the centroid of the segment and along the segment no matter the orientation of the segment. The angle between this principal axis and the *Y* axis of the camera frame is used to rotate the segment by bringing them into coincidence. At this step, the rotation is not completely removed as the angle around the *Y* axis between the direction the posture is facing and the one it should be facing (the *Z* axis of the camera frame) is not yet evaluated. To measure the latter, the centroid of the hand segment is determined. All points within 3 cm of the centroid are selected and assumed to belong to the hand palm. Using least square regression, a plane is fitted within the obtained set of points. The perpendicular direction to this plane is supposed to be the direction the posture is facing. An example is provided in Figure 11 where the original hand segment, the result of a random rotation applied to it, the result of the first rotation removal as well as the final result are shown.

**Figure 12.**
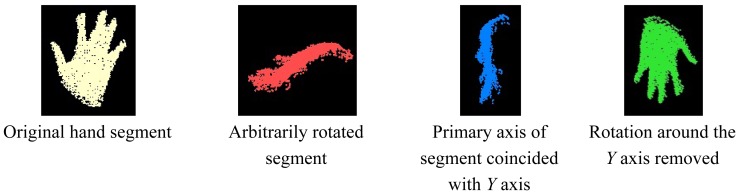
Example of an incorrect rotation removal of a hand segment.

One ambiguity appears in this methodology for evaluating the rotation of the hand segment: The orientations of the two axes determined. For example, after the first rotation removal, it is not always clear whether the hand segment is pointing upwards or downwards. The same problem has been notated for the second rotation removal. It has not been possible to rigorously solve this ambiguity and as a consequence for both rotation axes, the two orientations have to be considered every time; which results in four different possibilities for the un-rotated hand segment. An example of an incorrect, un-rotated hand segment that justifies this chosen solution is provided in Figure 12.

## 9. Experimental Results and Analysis

**Figure 13.**
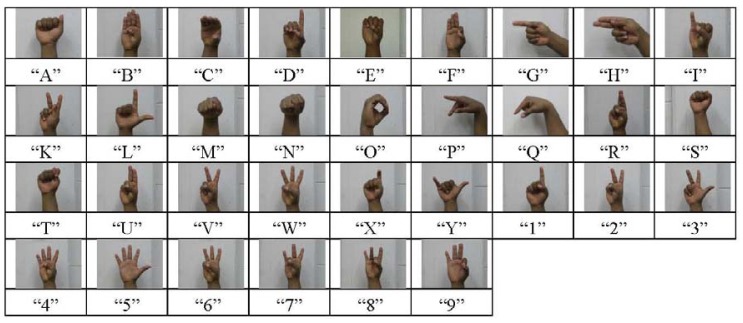
The 33 considered gestures (Dynamic gestures J and Z are out of scope of this study).

To evaluate this signature in combination with the algorithm for rotation removal, only 12 postures out of the 33 (Figure 13) have been considered. The main reason is to avoid postures that look alike and might cause some misclassifications. For example, Figure 13 shows the resemblance between “A”, “M”, “N”, “S” and between “T” and “E”. The same observation can be made with “D” and “1”, “W” and “6” or with “G” and “H”. In this case, only one training image has been used per posture while more than 1,000 images on average were tested for each selected posture.

The images have been collected from the same user. The range camera with its ability to collect at video rates has been used to capture the scene where the user performing one posture at a time was moving his hand in all three directions of the camera frame and rotating his hand meanwhile. The data collection was stopped once at least 1,000 images were captured for every single posture. For every posture, the set of images collected contained images where a 3D rotation has been applied to the hand movement.

**Figure 14.**
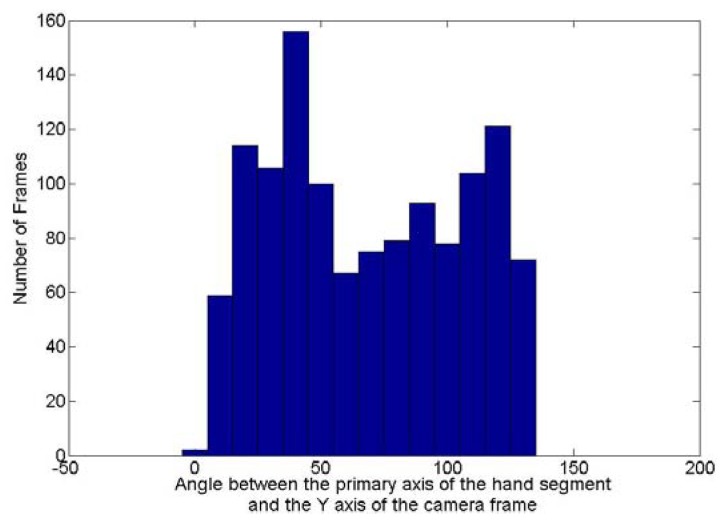
Values of angles between the primary axis of the hand segment and the Y axis of the camera for the frames acquired for the posture “5”.

**Figure 15.**
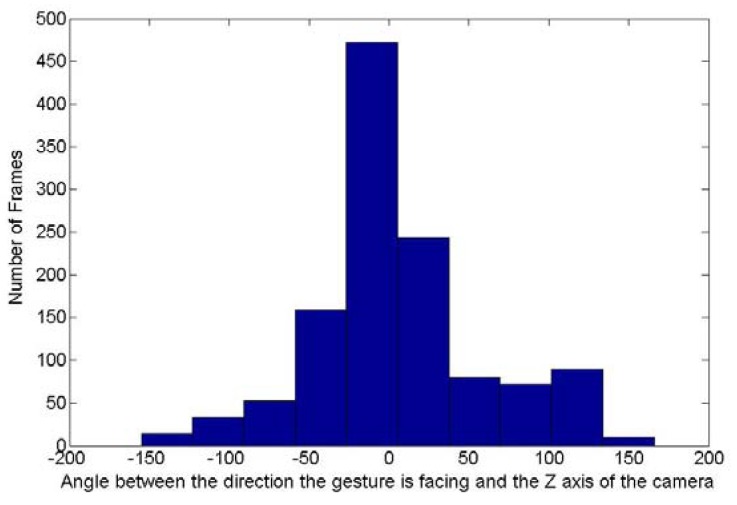
Values of angles between the direction the hand segment is facing and the Z axis of the camera for the frames acquired for the posture “5”.

To evaluate how rotated are the hand postures in the testing dataset, the rotation invariance algorithm has been applied to the images collected with the posture “5”. The purpose of this algorithm is to evaluate the two angles required to remove the orientation of the posture. Figures 14 and 15 show respectively the histogram of the occurrence of the angle between this principal axis and the Y axis of the camera frame and the one around the Y axis between the direction the posture is facing and the Z axis of the camera frame. Theoretically the first angle can fluctuate between 0 and 180° while the second one can vary between −180 and 180°. Figure 14 shows that for the frames collected with the posture “5”, the first angle varies between 0 and 130°. Figure 15 shows that the second angle varies between −150 and 150°. It can thus been concluded that there is a high variation of rotation in the dataset collected for the posture “5” and extend this conclusion to all the testing dataset as similar demonstrations could be done for all other postures tested.

**Figure 16.**
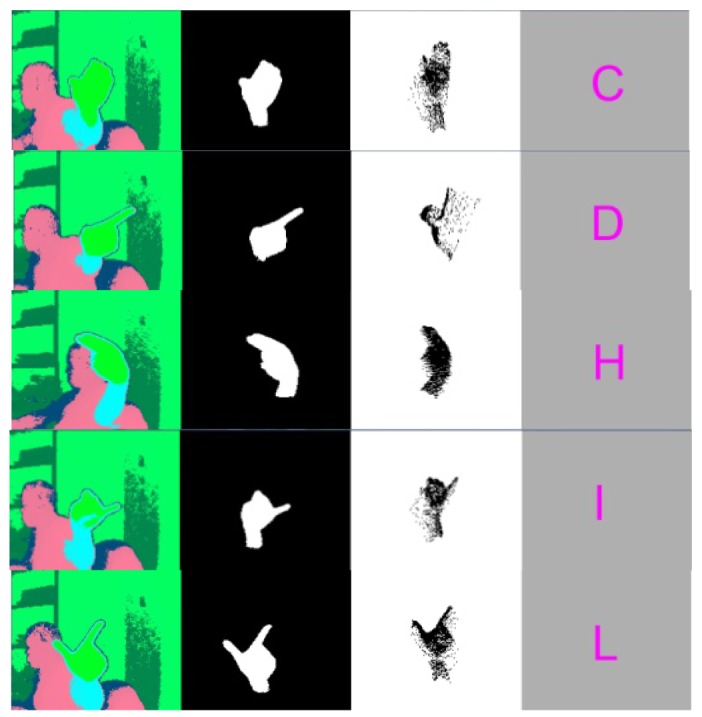

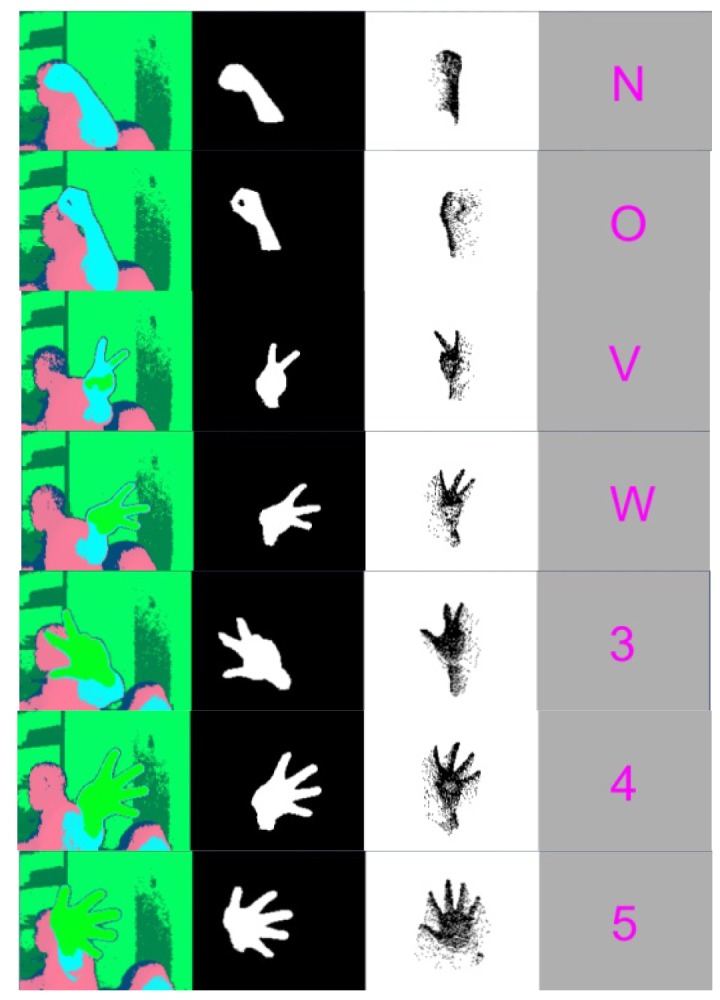
Considered postures Snapshots from real-time application showing the original range image, the segmented hand blob, the un-rotated segment and the recognized hand postures.

**Figure 17.**
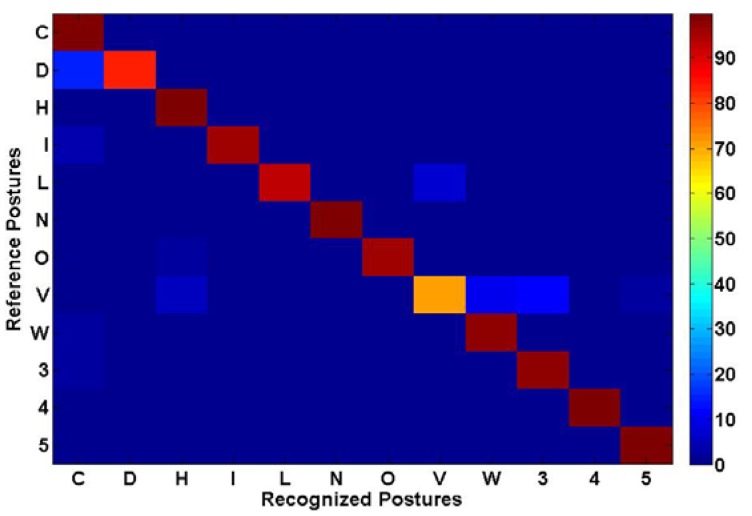
Confusion matrix.

**Figure 18.**
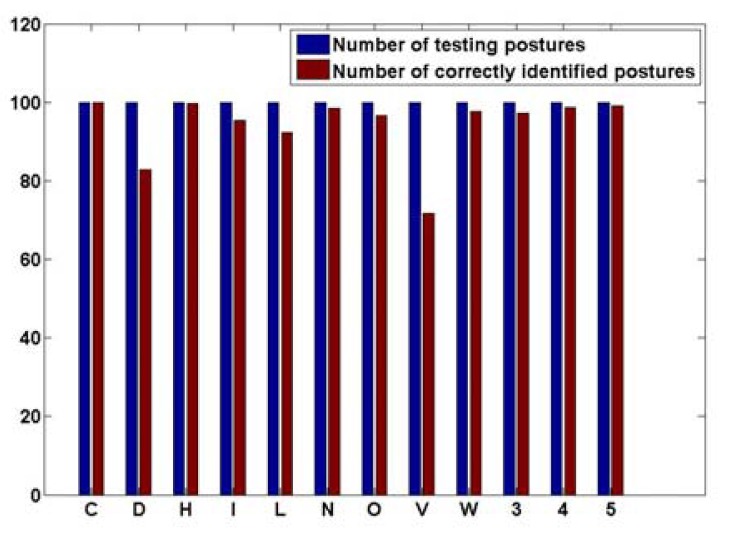
Recognition rates.

Some snapshots from the real-time application showing the original range image, the segmented rotated hand blob, the un-rotated segment as well as the recognized hand postures are presented in Figure 16. Figures 17 and 18 present, respectively, the confusion matrix and the recognition rates. Because of the careful selection of the postures that avoids any resemblance, the overall recognition rate is 93.88%. Very few mismatches have been noted, as shown by the Figure 17. This result is similar to the one obtained using the same methodology but with a different dataset that was described in the conference paper [38] where 98.24% overall recognition rate was obtained.

## 10. Comparison with Existing Methods

In [39], the authors have performed a comparative study where different shape descriptors for hand posture recognition have been evaluated using different classification methods. Using a database made of 11 postures and 1,000 images per posture taken from different users, the hand posture recognition has been performed with Hu-moments, Zernike moments and Fourier descriptors as features and Bayesian classifier, Support vector machine, k-Nearest Neighbors (k-NN) and Euclidian distance as classifiers. The best result achieved is 87.9% using the k-NN and Fourier descriptors. Another study, [40] shows 88.8% overall recognition rate after testing 12 postures with 20 images for each resulting in 240 tested images. In this study, the authors use the orientation histogram as feature and the posture matching has been accomplished using Normalized Cross Correlation. Table 3 summarizes the different aspects considered in the comparison.

Though the methodology described herein is tested on similar number of postures, it provides a higher recognition rate and has several advantages over these methods: the use of 3D images, a segmentation process independent on the skin of the colour, on the background of the image and on whether the user needs to wear long sleeves or not. In addition, only one training image is required per posture compared to 500 in [40] corresponding to 50% of the dataset.

## 11. Conclusions and Future Work

The current paper addresses the following question: How to recognize hand postures independently of the hand orientation while using a better representation of the hand compared to the mostly available ones in literature? Though simplistic, the proposed signature associated with the rotation invariant algorithm has been successful in recognizing 12 postures taken from the American Sign Language alphabet. Indeed, 93.88% of the 14,732 postures tested have been correctly recognized. This method uses a 3D representation of the hand and it has been proven the robustness of the rotation invariant algorithm. In addition, the objective was to design a real-time application and thus reduce as much as possible the recognition process time. To achieve the latter, only one training image has been considered in the supervised classification.

In future work, the focus will be made on the 3D signature by improving the alignment of two hand postures before their comparison in order to always compare corresponding parts of the hand posture. To be able to achieve the final goal being the recognition of all the 33 postures appearing in the alphabet of the American Sign Language, an improvement of the noise removal is also required. Furthermore, dynamic gestures involving one or two hands and also multiple cameras will be addressed.

## Figures and Tables

**Table 1.** Evaluation of the time required for a segmentation of an acquired image.

	**Range-based segmentation (s)**	**Noise Removal (s)**	**Connected component analysis (s)**	**Total Time (s)**
**Minimum**	1.10	0.00	0.10	1.30
**Average**	4.00	0.10	1.91	5.81
**Maximum**	2.01	0.00	0.53	2.55
**Standard deviation**	0.39	0.02	0.32	0.67

**Table 2.** Evaluation of the tracking accuracy in cm.

	**RMS(*X*)**	**RMS(*Y*)**	**RMS(*Z*)**
**Kalman Filter Tracking**	0.3	0.5	0.8
**Mean-Shift Tracking**	0.3	0.6	0.7

**Table 3.** Comparison of methods and results between the current study and two others.

**Postures considered**	**Taken from the alphabet of the American sign language**	**Taken from the alphabet of the American sign language**	**Taken from the alphabet of the American sign language**
Number of postures considered	10	12	12
Number of testing images per posture	1,000	20	1,000
Features used	Fourier descriptors	Oriented gesture descriptors	3D signature
Classifier used	k-NN	Normalized cross correlation	k-NN
Number of templates per posture	500	Not Provided	1
Number of users for the testing database	Multiple users	Not Provided	1 user
Overall recognition rate	87.9%	88.8%	93.88%
